# Activated TAZ induces liver cancer in collaboration with EGFR/HER2 signaling pathways

**DOI:** 10.1186/s12885-022-09516-1

**Published:** 2022-04-19

**Authors:** Hyuk Moon, Hyunjung Park, Min Jee Chae, Hye Jin Choi, Do Young Kim, Simon Weonsang Ro

**Affiliations:** 1 Department of Genetics and Biotechnology, College of Life Sciences, Kyung Hee University, 17104 Yongin-si, Gyeonggi-do Korea; 2 Division of Medical Oncology, Department of Internal Medicine, Yonsei University College of Medicine, 03722 Seoul, South Korea; 3 Yonsei Liver Center, Severance Hospital, 03722 Seoul, South Korea; 4 Department of Internal Medicine, Yonsei University College of Medicine, 03722 Seoul, South Korea

**Keywords:** Hepatocellular carcinoma, Cholangiocarcinoma, TAZ, Hydrodynamic transfection, EGFR/HER2

## Abstract

**Background:**

Liver cancer is a major global health concern due to the steady increases in its incidence and mortality. Transcription factors, yes-associated protein (YAP) and WW domain-containing transcription regulator protein 1 (WWTR1, also known as TAZ) have emerged as critical regulators in human hepatocellular carcinoma (HCC) and cholangiocarcinoma (CC), the two major types of primary liver cancer. However, our study as well as other previous reports have shown that activation of YAP and TAZ (YAP/TAZ) in adult murine livers is insufficient for the development of liver cancer, suggesting a requirement for an additional oncogenic collaborator for liver carcinogenesis in adulthood. Therefore, we sought to identify the oncogenic partners of YAP/TAZ that promote hepatocarcinogenesis in adults.

**Methods:**

Data analysis of the transcriptome of patients with liver cancer was performed using the national center for biotechnology information (NCBI) gene expression omnibus (GEO) database and the cancer genome atlas (TCGA). The cancer therapeutics response portal (CTRP) was used to investigate the correlation between sensitivity to chemicals and the copy number of TAZ in human cancer cell lines. Transposons encoding constitutively activated forms of TAZ (TAZ^S89A^), BRAF (BRAF^V600E^), and PIK3CA (PI3K^E545K^) were used for hydrodynamic tail vein injection. Mice were monitored at least twice per week and sacrificed when moribund. Tumor-bearing livers were formalin fixed for hematoxylin–eosin staining and immunohistochemistry.

**Results:**

Through database analyses, we identified EGFR/HER2 signaling to be essential in human cancers with high TAZ activity. Furthermore, immunohistochemical analyses showed that human HCC and CC tissues with high YAP/TAZ activities exhibited concomitant activation of EGFR/HER2 signaling pathways. To demonstrate that EGFR/HER2 signaling promotes YAP/TAZ-mediated hepatocarcinogenesis, TAZ^S89A^ was simultaneously expressed in murine adult livers with BRAF^V600E^ or PI3K^E545K^, activated forms of effector molecules downstream of EGFR/HER2 signaling pathways. Expression of TAZ^S89A^ plus BRAF^V600E^ induced HCC, whereas TAZ^S89A^ and PI3K^E545K^ led to the development of CC-like cancer.

**Conclusions:**

Our study demonstrates that TAZ collaborates with EGFR/HER2 signaling pathways to induce both HCC and CC.

**Supplementary information:**

The online version contains supplementary material available at 10.1186/s12885-022-09516-1.

## Background

Liver cancer is the fourth most common cancer worldwide, and its incidence and mortality rates have been increasing steadily [1, 2]. Liver cancer typically exhibits heterogeneous histological features and poor prognosis [3, 4]. The most common type of primary liver cancer is hepatocellular carcinoma (HCC), which accounts for about 80% of the cases, followed by cholangiocarcinoma (CC), which contributes to 10–20% of primary liver cancers [1, 5]. Although the two types of liver cancers have fundamentally different molecular and clinical characteristics, they share overlapping risk factors and oncogenic signaling pathways. Moreover, adult hepatocytes have been identified as the origin of both HCC and CC in recent studies [6–8].

The Hippo signaling pathway consists of a kinase cascade that regulates a variety of cellular processes [9–11]. The core kinases include the mammalian STE20-like protein kinase 1 and 2 (MST1 and MST2, also known as STK4 and STK3, respectively), that phosphorylate the large tumor suppressor 1 (LATS1) and 2 (LATS2). When Hippo signaling is activated, LATS1 and LATS2 (LATS1/2) phosphorylate yes-associated protein (YAP) and WW domain-containing transcription regulator protein 1 (WWTR1, also known as TAZ), thereby inhibiting the nuclear import of YAP and TAZ (YAP/TAZ). In contrast, when Hippo signaling is inactivated, unphosphorylated YAP/TAZ is transported into the nucleus, resulting in the transcriptional activation of a plethora of genes involved in cell proliferation and survival through interaction with the TEA domain family members (TEADs) [12–14]. Recent years have witnessed significant advances in our understanding of the roles of YAP/TAZ in HCC and CC [15–17]. Knockouts of the key mediators of the Hippo signaling pathway in embryos developed HCC and CC in adult mice [18–21]. Likewise, prolonged overexpression of YAP/TAZ from neonatal stages induced HCC later in adulthood [22, 23]. In contrast to the prolonged activation of YAP/TAZ from embryonic stages or starting at birth, activation of YAP in the adult liver generally failed to induce cancer, but caused hepatomegaly or preneoplastic lesions [22, 24]. This suggests that activation of YAP or TAZ alone, is insufficient to induce cancer in adult livers, and requires additional oncogenic partners. Liver cancers often show dysregulation of multiple signaling pathways such as RAS-RAF-MEK-ERK, PI3K-AKT, Wnt/β-catenin, and hedgehog signaling pathways [25, 26]. Concurrent alterations in multiple signaling pathways in liver cancer suggest that oncogenic collaborations may be required to initiate or promote tumor development in the liver. In this study, we used database analyses and murine liver-specific transgenic models to identify the oncogenic partners of TAZ that promote tumorigenesis in the liver.

## Methods

### Data analyses from publicly available databases

Data analysis of the transcriptome of patients with liver cancer was performed using the following publicly available databases: National center for biotechnology information (NCBI) gene expression omnibus (GEO) database (Accession Nos. GSE36376, GSE26566, GSE64041, and GSE32958) and the cancer genome atlas (TCGA) projects TCGA-LIHC (liver hepatocellular carcinoma) and TCGA-CHOL (cholangiocarcinoma).

### Gene set enrichment analysis (GSEA)

Gene sets were downloaded from the molecular signatures database (MSigDB) (http://software.broadinstitute.org/gsea/) of the Broad Institute (Cambridge, MA, USA). Using the gene set permutation, the signal-to-noise ratio of the genes was used to determine the statistical enrichment of gene sets.

### Animal experiments

Wild-type (C57BL6/N) male mice were purchased from Orientbio (Seongnam, Korea), maintained in a specific pathogen free (SPF) facility under a 12 h light/dark cycle, and provided food and water ad libitum. All experiments using mice were approved and done in accordance with the Institutional Animal Care and Use Committee of The Yonsei University College of Medicine under protocol IACUC #2015 − 0410. The studies were carried out in compliance with ARRIVE guidelines.

### Recombinant DNA

Transposons encoding the constitutively activated forms of TAZ (pT3/EF5a TAZS89A) was a kind gift from Dr. Xin Chen at the University of California, San Francisco. The plasmids pT2/EGFP and pPGK-SB13 were described previously [27]. Open reading frames (ORFs) encoding the activated forms of BRAF (BRAF^V600E^) and PIK3CA (PI3K^E545K^) were polymerase chain reaction (PCR)-amplified from pBabe-B-RAF-V600E (#17,544; Addgene, Watertown, MA, USA) and pBabe-puro-HA-PIK3CA-E545K plasmids (#12,525; Addgene), respectively. These amplified PCR products were cloned into pT2/BH transposon vectors (#26,556; Addgene) to generate the plasmids, pT2/BRAF^V600E^, and pT2/PI3K^E545K^, respectively.

### Hydrodynamic tail vein injection (HTVI)

Mice were randomly assigned to experiments. For hydrodynamic tail vein injection, DNA mixtures containing transposons (pT2- or pT3- plasmids) and transposase-encoding plasmids (pPGK-SB13) were prepared using the Endo-Free Maxi Kit (Qiagen, Hilden, Germany). The DNA plasmids were diluted in lactated Ringer’s solution, and then injected into the lateral tail veins of male mice (5–6 weeks old; 0.1 mL/g body weight) within 7 s, as previously described [28]. For single transgenic livers, mouse of 20 g body weight received 24 µg of pT3/EF5a TAZS89A transposons (or pT2/EGFP transposons as a control) and 9 µg of pPGK-SB13 plasmids via HTVI. In experiments investigating oncogenic collaboration between TAZ and EGFR/HER2 downstream signaling pathways, 12 µg of pT3/EF5a TAZS89A transposons and 9 µg of pPGK-SB13 plasmids were mixed with 12 µg of pT2/ BRAF^V600E^, pT2/ PI3K^E545K^ or pT2/EGFP transposons, and the DNA mixtures were used for HTVI.

### Cancer cell line (CCL) sensitivity analysis for compounds

The cancer therapeutics response portal (CTRP) v2 (http://www.broadinstitute.org/ctrp.v2.2) was used to investigate the correlation between sensitivity to chemicals and the copy number of TAZ in the CCLs. The CTRP v2 contains 860 CCLs, of which 827 are characterized as part of the cancer cell line encyclopedia (CCLE), and represent 25 different lineages. The data included a 72 h, 16-point dose-response screen to assess the sensitivity of these cell lines to a total of 481 compounds, including 70 FDA-approved drugs, 100 candidate compounds, and 311 small molecules [29, 30].

### Histology and IHC

Liver tissue samples were fixed in 10% neutral-buffered formalin and embedded in paraffin. The paraffin sections were deparaffinized in xylene and rehydrated by passing through gradually decreasing strengths of ethanol. The sections were then stained with hematoxylin and eosin (H&E) and standard IHC for histopathological analysis. IHC staining was conducted using antibodies against pan CK (1:100; ab234297; Abcam, Cambridge, UK), SOX9 (1:400; #82,630; Cell Signaling Technology, Danvers, MA, USA), Ki-67 (1:400; ab15580; Abcam), YAP (1:100; ab52771; Abcam), TAZ (1:100; #72,804; Cell Signaling Technology), phospho-EGFR (Phospho-Y1068; 1:100; ab40815; Abcam), phospho-MEK1/2 (phospho-Ser217/221; 1:200; #9154; Cell Signaling Technology), phospho-AKT (Phospho-Ser473; 1:400; ab81283; Abcam), phospho-STAT3 (Phospho-Y705; 1:100; #9145; Cell Signaling Technology), HNF4α (1:200; #3113; Cell Signaling Technology), and NOTCH2 (1:100; #5732; Cell Signaling Technology). After incubation with the primary antibodies, the sections were incubated with the appropriate biotinylated secondary antibodies, followed by treatment with freshly prepared DAB substrates (Vector Laboratories, Burlingame, CA, USA).

### Protein extraction and western blotting

Frozen mouse liver samples were homogenized and digested in 1× RIPA lysis buffer containing complete protease and phosphatase inhibitor cocktails (P3200; GenDEPOT, Barker, TX, USA). Proteins were separated by sodium dodecyl sulfate polyacrylamide gel electrophoresis (SDS-PAGE) and transferred onto a polyvinylidene difluoride (PVDF) membrane. The membranes were immunoblotted with antibodies against phospho-AKT (#4060; Cell Signaling Technology), phospho-MEK1/2 (#9154; Cell Signaling Technology), NFκB p65 (sc-372; Santa Cruz Biotechnology, Dallas, TX, USA), NOTCH1 (ab8925; Abcam), and GAPDH (#2118; Cell Signaling Technology). Finally, the immunoreactive proteins were detected using the West-Q Pico Dura ECL Solution (W3653; GenDEPOT, Barker, TX, USA).

### Human liver tissue specimens

Human primary HCC and CC tissues were obtained from the biobank at the Severance Hospital, Seoul, Korea. Tissues were collected immediately following surgery and stored at -80 °C until processing and use. This study was approved by the Independent Institutional Review Board of Severance Hospital (IRB number: 4-2018-1087) and conformed to the ethical guidelines of the Declaration of Helsinki (1975).

### Statistical analysis

Statistical analyses were carried out with two-tailed unpaired *t*-tests using GraphPad Prism Software (GraphPad, La Jolla, CA, USA). All values are expressed as means. Significant differences between two groups are denoted by asterisks (*, P < 0.05; **, P < 0.01; ***, P < 0.001).

## Results

### Activation of YAP/TAZ signaling in human liver cancer

**Fig. 1:**
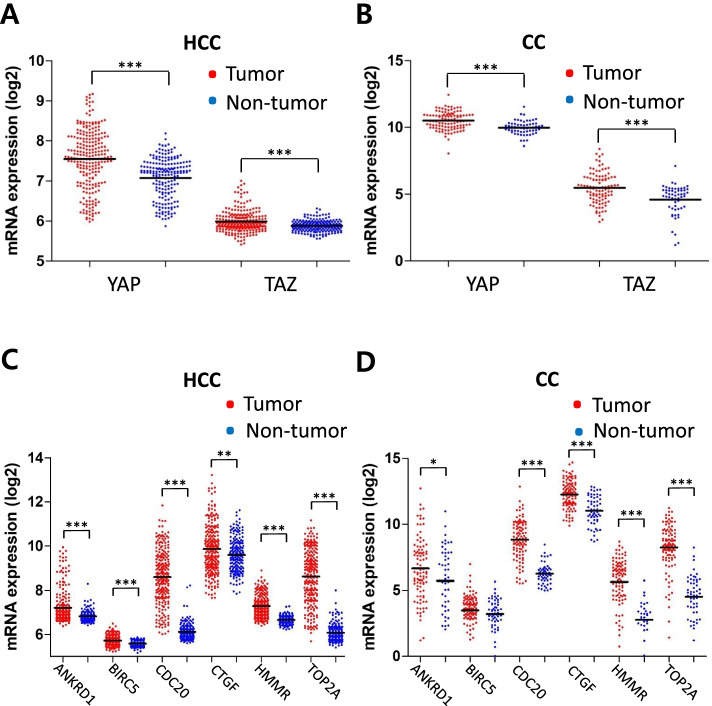
Activation of YAP/TAZ signaling in human liver cancers. **A**,** B** Expression levels of YAP and TAZ (WWTR1) in tumor and non-tumor counterparts were compared using gene expression omnibus (GEO) database for HCC (**A**) and CC (**B**). **C**,** D** Expression levels of YAP/TAZ target genes in tumor and non-tumor counterparts were compared using the same database for HCC (**C**) and CC (**D**). Mean expression level of each group is indicated with horizontal lines. (*, *P <* 0.05; **, *P <* 0.01; ***, *P <* 0.001)

First, we investigated whether YAP/TAZ activity was elevated in human liver cancer using GSE datasets (GSE36376 for HCC and GSE26566 for CC). Expression levels of YAP and TAZ (YAP/TAZ) were significantly higher in HCC (*n* = 240) compared with those in non-tumor tissue (*n* = 193) (Fig. 1A). Similarly, overexpression of YAP/TAZ was observed in CC (*n* = 104) when compared to that in matching non-tumor hepatic tissues (*n* = 59) (Fig. 1B). In line with the findings, direct YAP/TAZ target genes such as ANKRD1 (ankyrin repeat domain-containing protein 1) and CTGF (connective tissue growth factor) were found significantly upregulated both in HCC and CC (Fig. 1C and D). Similar findings were observed when other GSE datasets were analyzed, such as GSE64041 (for HCC, *n* = 120) and GSE32958 (for CC, *n* = 23) in which gene set enrichment analysis (GSEA) showed significant enrichments of YAP/TAZ signature gene sets in HCC and CC (Supplementary Fig. 1).

### TAZ induces proliferation and de-differentiation of hepatocytes

**Fig. 2:**
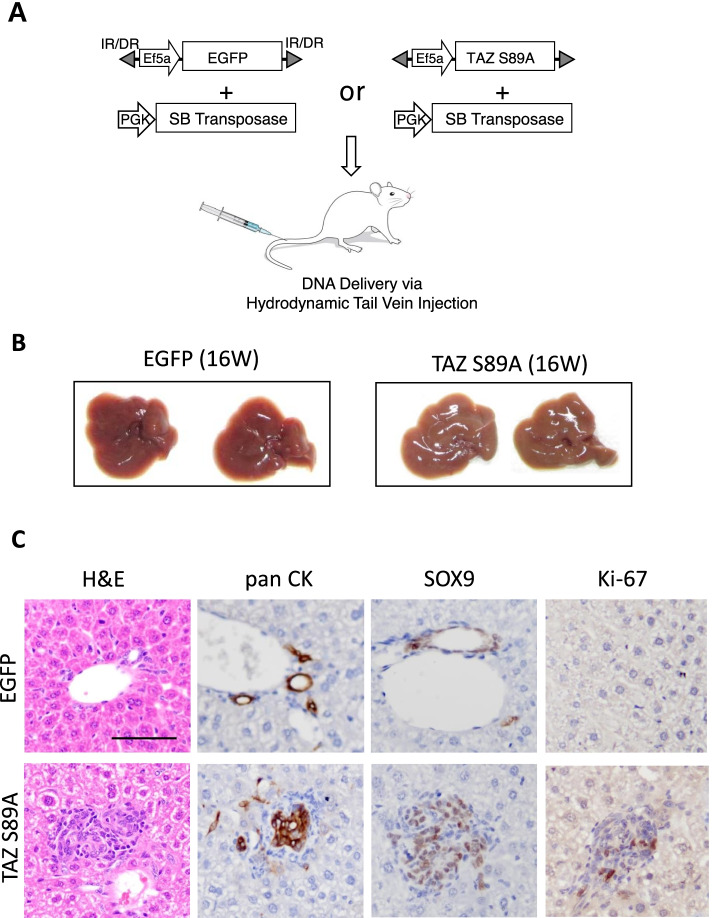
TAZ alone does not induce cancer. **A** Schematic illustration of the experimental procedure. Transposons encoding TAZ^S89A^ were mixed with plasmids expressing the *Sleeping Beauty* transposase, and injected via hydrodynamic tail vein injection. **B** Gross morphology of representative livers expressing TAZ^S89A^ and enhanced green fluorescent protein (EGFP, a control). Livers were harvested at 16 weeks following the hydrodynamic injection (*n* = 5). **C** Microscopic images showing H&E and IHC staining for pan CK, SOX9, and Ki-67 in sections of livers shown in (**B**). Scale bar, 50 μm

Given that YAP/TAZ activity was significantly upregulated in liver cancers, we tested oncogenic potentials of YAP/TAZ in murine livers using a liver-specific transgenic approach [28]. Based on previous reports that a constitutively active form of human YAP (YAP^S127A^) induced proliferation and dedifferentiation of hepatocytes [24, 31], we questioned whether its paralogue, TAZ could also exert a similar effect on hepatocytes. Transposons encoding an active form of human TAZ (TAZ^S89A^) were hydrodynamically delivered to the liver together with plasmids encoding the *Sleeping Beauty* transposase (Fig. 2 A). Although expression of TAZ^S89A^ in the liver did not induce visible tumors when examined at 16 weeks following the injection, microscopic examination of liver tissues from TAZ^S89A^ mice revealed small hyperplastic lesions (Fig. 2B and C). Proliferation of hepatic cells in the lesions was verified using Ki-67 staining, which was not present in control livers transfected with transposons expressing enhanced green fluorescent protein (EGFP). Further, cells in the lesions expressing TAZ^S89A^ were stained positive for pan CK and SOX9, molecular markers for liver progenitor cells (Fig. 2 C). In contrast, location of cells positive for pan CK and SOX9 staining was confined to hepatic ducts in control livers. The findings indicate that TAZ induces hepatic proliferation and dedifferentiation.

### EGFR/HER2 signaling as a putative oncogenic collaborator of TAZ in liver

**Fig. 3:**
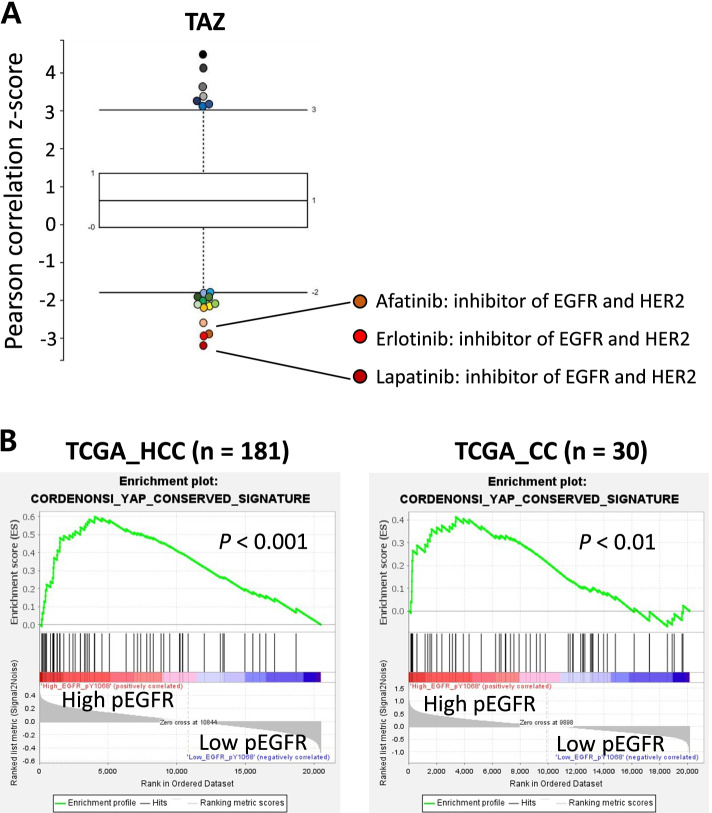
EGFR/HER2 signaling pathways are associated with TAZ in human cancer.** A** Correlation between chemical sensitivity and copy number of TAZ gene in human cancer cell lines. Y-axis indicates Z-scored Pearson correlations. Box plot represents Tukey outliers. **B** Correlation between the YAP/TAZ signals and the levels of phosphorylated EGFR (EGFR _pY1068). GSEA was performed with regard to levels of phosphorylated EGFR using the TCGA database. Barcode indicates gene positions and the y-axis indicates the extent of enrichment

Because TAZ^S89A^ alone was insufficient to induce liver cancer *in vivo*, we speculated that an oncogenic collaborator is required for TAZ-mediated liver carcinogenesis in adult livers. To identify signaling pathways that are closely associated with TAZ in human carcinogenesis, we took advantage of the sensitivity analyses of compounds in human cancer cell lines (CCLs) available online through the Broad Institute (http://www.broadinstitute.org). We conducted a correlation-based analysis of 860 human CCLs with 481 compounds through the cancer therapeutic response portal (CTRP v2). The analysis identified lapatinib, erlotinib, and afatinib as the most lethal compounds for cell lines with a high copy number of the TAZ gene (Fig. 3 A). All three chemical compounds are inhibitors of receptor tyrosine kinases (RTKs), mainly targeting EGFR and HER2. As the data showed that cancer cells with a high copy number of the TAZ gene were sensitive to EGFR/HER2 blockade, we speculated that the EGFR/HER2 signaling pathways may be required for the survival of cancer cells with a high TAZ activity, or possibly that activation of EGFR/HER2 signaling may be required for TAZ-mediated carcinogenesis.

To verify associations between TAZ signals and activation of EGFR/HER2 signaling pathways in liver cancers, we performed both database and immunohistochemistry (IHC) analyses. Binding of its ligands to EGFR leads to phosphorylation at multiple tyrosine residues in the cytoplasmic tails of the receptors, triggering activation of downstream signaling cascades such as RAS-RAF-MEK-ERK, PI3K-AKT, and JAK-STAT3 signaling pathways. Of note, phosphorylation at the Y1068 of EGFR recruits the Grb2/Shc/SOS adapter complex to the plasma membrane and subsequently activates the RAS-RAF-MEK-ERK signaling pathway. GSEA using the TCGA database showed enrichments of YAP/TAZ signature gene sets in liver cancers with high levels of phosphorylated EGFR (pEGFR) (Fig. 3B). Further, activation of YAP/TAZ signaling was significantly correlated with elevated levels of phosphorylated ERK (pERK), a downstream effector molecule of EGFR-RAS signaling (Supplementary Fig. 2). The database analyses signify associations between YAP/TAZ and EGFR/HER2 signaling pathways both in human HCC and CC.

**Fig. 4:**
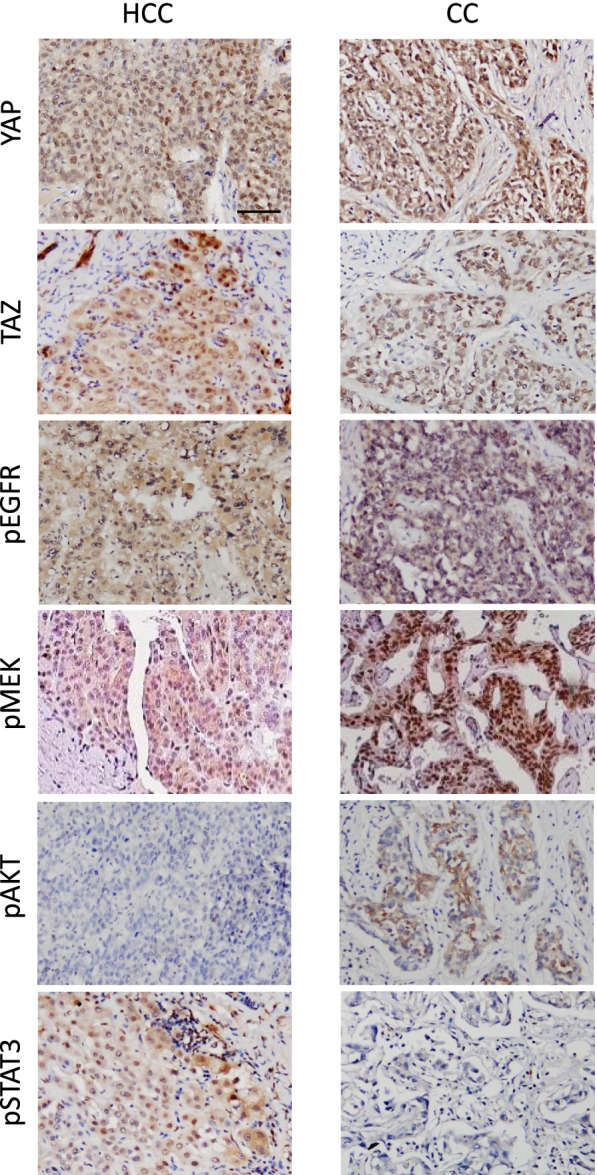
Activation of YAP/TAZ and EGFR/HER2 signaling pathways in human liver cancers. Microscopic images showing IHC staining for YAP, TAZ, pEGFR, and phosphorylated downstream effector molecules of EGFR/HER2 in human HCC and CC. Scale bar, 50 μm

In line with the findings, IHC analysis of human HCC and CC with a high YAP/TAZ activity showed elevated levels of phosphorylation in EGFR, as well as increased phosphorylation in EGFR downstream molecules such as MEK, AKT, and/or STAT3, suggesting a concomitant activation of YAP/TAZ and EGFR signaling pathways in liver cancers (Fig. 4 and Supplementary Table 1). About 87% of human liver cancer with high YAP/TAZ activity (26 out of 30 cancer samples) showed concomitant activation of the downstream signaling pathways of EGFR/HER2 (Supplementary Table 1). In contrast, 11% of liver cancer samples with minimal YAP/TAZ activity (one out of nine cancer samples) revealed activation of the EGFR/HER2 signaling pathways. Thus, both the database and IHC analyses indicated a strong association between YAP/TAZ and EGFR/HER2 downstream signaling pathways in human hepatocarcinogenesis.

### TAZ induces cancer through the cooperation with RAS and PI3K signaling pathways

**Fig. 5:**
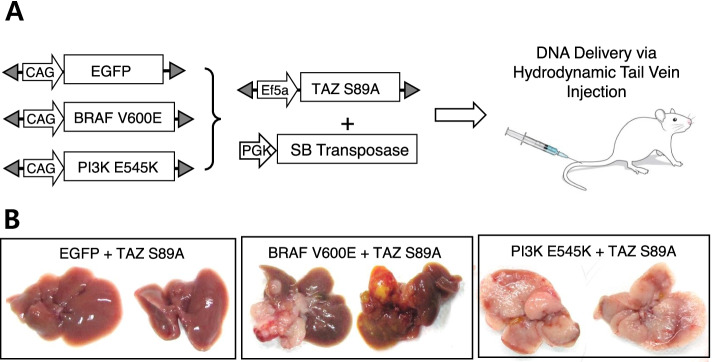
TAZ^S89A^ induces liver cancer in collaboration with either BRAF^V600E^ or PI3K^E545K^. **A** Schematic illustration of the experimental procedure. **B** Gross morphology of representative livers expressing EGFP (control), BRAF^V600E^, and PI3K^E545K^, respectively, together with TAZ^S89A^. Livers were harvested at 6 weeks following the hydrodynamic injection (*n* = 5 mice per group)

**Fig. 6:**
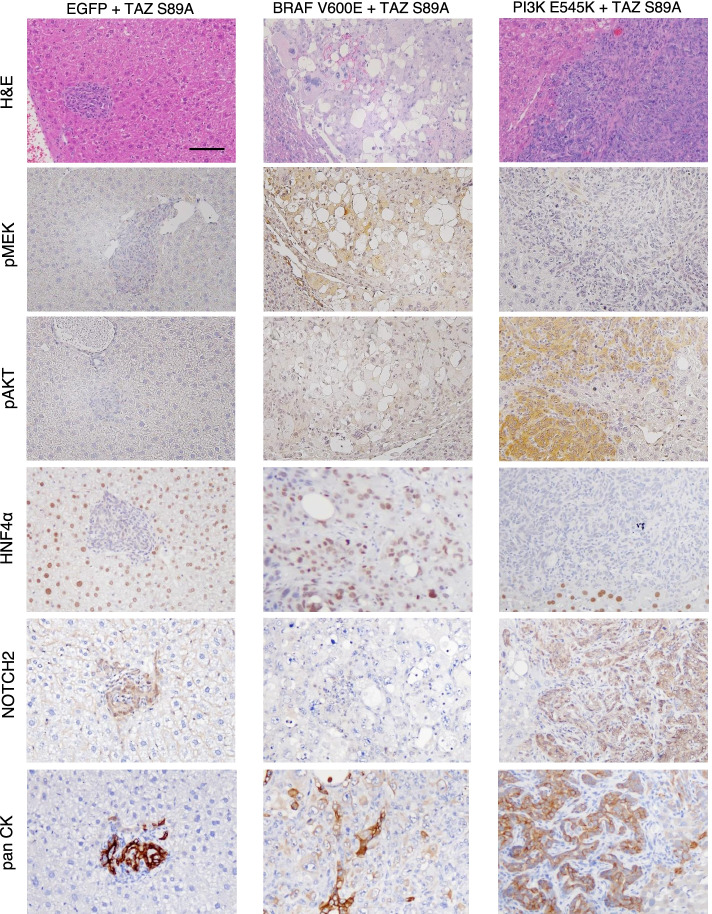
Histological analyses of tumors induced by TAZ^S89A^ plus BRAF^V600E^ and TAZ^S89A^ plus PI3K^E545K^. H&E and IHC staining images of liver sections expressing indicated oncogenes. Scale bar, 50 μm

Based on these data, we tested whether the EGFR/HER2 downstream signaling pathways collaborated with TAZ to induce cancer in adult livers. Transposons encoding a constitutively active form of BRAF (BRAF^V600E^) were delivered to the liver together with those encoding TAZ^S89A^ via hydrodynamic tail vein injection (Fig. 5 A). Simultaneous expression of TAZ^S89A^ and BRAF^V600E^ in the liver induced cancer with an incidence rate of 100% at 6 weeks following the hydrodynamic injection (Fig. 5B). Likewise, co-expression of a constitutively active form of PIK3CA (PI3K^E545K^) with active TAZ induced liver cancer in all the mice tested, confirming that the PI3K-AKT signaling pathway also efficiently cooperates with TAZ to induce liver cancer. Microscopic examination of the liver cancers revealed that the TAZ^S89A^ plus BRAF^V600E^ tumors were HCC-like, while the TAZ^S89A^ plus PI3K^E545K^ tumors resembled CC (Fig. 6). In line with the microscopic tumor morphology, immunohistochemical analyses revealed nuclear expression of HNF4α, a representative hepatocytic marker, in tumors induced by TAZ^S89A^ plus BRAF^V600E^ while TAZ^S89A^ plus PI3K^E545K^ tumors exhibited high expression of Notch1 and Notch2, critical players in development of CC (Fig. 6 and Supplementary Fig. 3). Of note, tumors induced by TAZ^S89A^ plus PI3K^E545K^ showed high expression of pan CK, while TAZ^S89A^ plus BRAF^V600E^ tumors exhibited patched expression of the protein (Fig. 6).

## Discussion

YAP/TAZ signaling is involved in multiple processes during carcinogenesis, including promotion of cell proliferation, induction of tissue invasion of tumor cells, and maintenance of cancer stem cells [12]. Several oncogenic signaling pathways have been reported to crosstalk with YAP/TAZ in carcinogenesis, including the Wnt/β-catenin and LKB1 signaling pathways [32–34]. Notably, the involvement of YAP in KRAS-mediated neoplastic progression in pancreatic ductal adenocarcinoma (PDAC), underscores the potential crosstalk between YAP/TAZ and RAS signaling pathways in PDAC [35].

The RAS-RAF-MEK-ERK signaling pathway is activated via cell surface receptors such as EGFR, HER2, and platelet-derived growth factor receptor (PDGFR). Binding of ligands to these receptors leads to the activation of the cytoplasmic tyrosine kinases that phosphorylate tyrosine residues in the cytoplasmic tails of the receptors. This event recruits the Grb2/Shc/SOS adapter complex to the plasma membrane and subsequently converts membrane-tethered GDP-bound RAS to active GTP-bound RAS [36, 37]. Activated RAS triggers the mitogen-activated protein kinase signaling cascade through the RAF-MEK-ERK axis. The ligand-bound receptor tyrosine kinases also trigger the PI3K-AKT signaling axis via phosphorylation of tyrosine residues in the cytoplasmic tails [38, 39]. The RAS-RAF-MEK-ERK and PI3K-AKT signaling pathways are activated in approximately 50% of human liver cancers, implying their significant roles in hepatocarcinogenesis [40–44]. Another important downstream signaling pathway of EGFR/HER2 is JAK/STAT3. The JAK/STAT3 signaling pathway activates multiple target genes involved in cell proliferation, survival, stemness, and etc. [45]. The signaling pathway is a significant contributor to liver cancer development and targeting the JAK/STAT3 signaling pathway is proposed as a promising therapy for HCC [46, 47].

In this study, IHC analysis showed that RAS-RAF-MEK-ERK signaling was activated in 85% of human HCC with high YAP/TAZ activity, whereas PI3K-AKT signaling was active only in 23% of human HCC with high YAP/TAZ activity, suggesting that the RAS-RAF-MEK-ERK pathway is the major contributor to HCC development with YAP/TAZ activation (Supplementary Table 1). In line with the IHC results, co-expression of BRAF^V600E^ and TAZ^S89A^ induced HCC in the liver (Figs. 5 and 6). Of note, activation of PI3K signaling (through the expression of PI3K^E545K^) together with TAZ activation led to CC-like liver cancer, suggesting a role for PI3K-AKT signaling in the development of cholangiocarcinoma. Tumors induced by TAZ^S89A^ plus PI3K^E545K^ showed elevated expression of Notch1 and Notch2 [48, 49]. Of note, Wang et al. recently reported that genetic ablation of Notch2 suppressed development of CC phenotypes in murine livers expressing activated forms of AKT and YAP, while deletion of Notch1 did not affect CC development induced by AKT and YAP, signifying the role of Notch2 in hepatocyte-derived CC formation in mice [50].

Our transgenic liver cancer models induced by TAZ^S89A^ plus BRAF^V600E^ and TAZ^S89A^ plus PI3K^E545K^ are expected to be useful for various studies, such as investigation of molecular pathogenesis of liver cancers, cross-talks between YAP/TAZ and EGFR/HER2 signaling pathways, and etc. As well, the models can be effective applied to evaluate efficacies of molecular target therapies for HCC and CC. The applicability of the models for HCC and CC in preclinical studies will likely broaden in the future, considering simplicity of the methods in generating the models and significance of the oncogenic drivers in developing human HCC and CC.

## Conclusions

Our study identified RAS-RAF-MEK-ERK and PI3K-AKT signaling pathways as oncogenic collaborators of TAZ during liver carcinogenesis. This carcinogenic cooperation proposes a potential therapeutic strategy for the treatment of HCC and CC.

## Supplementary Information

**Additional file 1.**

**Additional file 2.**

**Additional file 3.**

## Data Availability

The datasets used and/or analyzed during the current study are available from the corresponding author upon reasonable request.

## Abbreviations

HCC: hepatocellular carcinoma
CC: cholangiocarcinoma
MST1 and MST2: mammalian STE20-like protein kinase 1 and 2
LATS1 and LATS2: large tumor suppressor 1 and 2
YAP: yes-associated protein
WWTR1: WW domain-containing transcription regulator protein 1
TEAD: TEA domain family member
GSEA: gene set enrichment analysis
CCL: cancer cell line
RTK: receptor tyrosine kinase
IHC: immunohistochemistry
