# Supplementary material for: Activated TAZ induces liver cancer in collaboration with EGFR/HER2 signaling pathways
Source: BMC Cancer. 2022 Apr 19;22:423. doi: 10.1186/s12885-022-09516-1 (PMC9019950; doi:10.1186/s12885-022-09516-1)
Supplement: Supplementary file 1 — Additional file 1. [file 12885_2022_9516_MOESM1_ESM.pdf]

Supplementary Figure 1

HCC CC

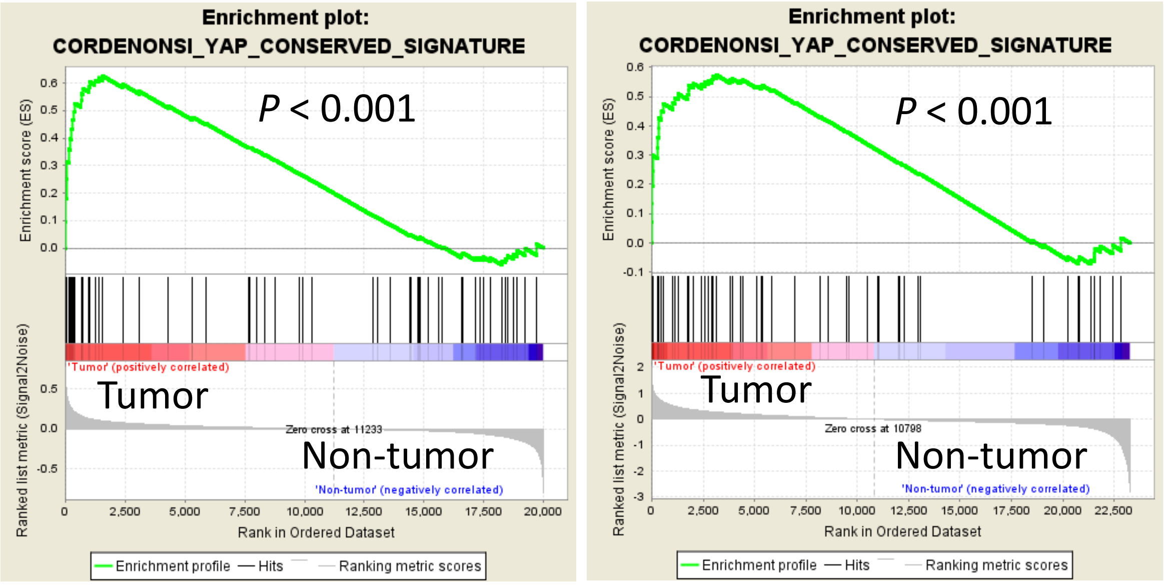

	Tumor_vs_Non-tumor	
DataSet	GSE64041 (HCC)	GSE32958 (CC)
GENESET	CORDENONSI_YAP_CONSERVED_SIGNATURE	
SIZE	57	57
NES	1.9171263	1.9856614
NOM p-val	0	0
FDR q-val	0	0

NES, normalized enrichment score; FDR, false discovery rate.

Supplementary Figure 1. Enrichment plots from gene set enrichment analysis (GSEA). YAP/TAZ signals are highly enriched both in HCC and CC (P < 0.001). Barcode indicates gene positions and the y-axis indicates the extent of enrichment.

Supplementary Figure 2

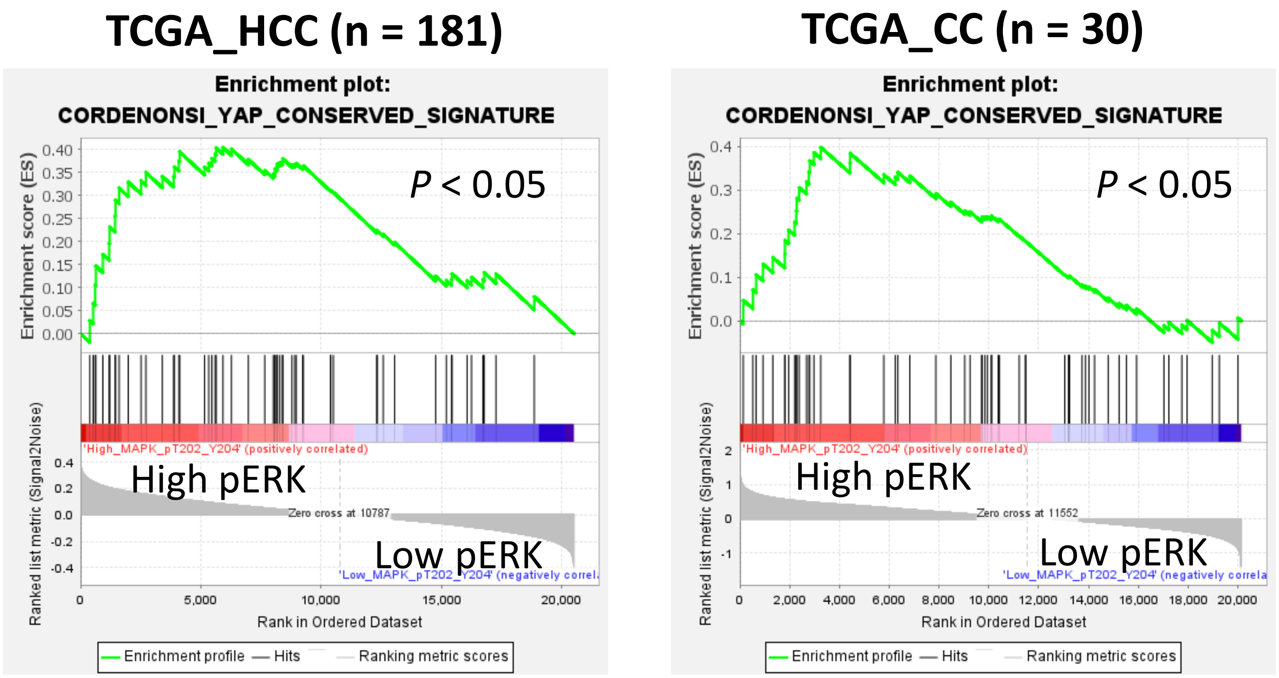

Supplementary Figure 2. Correlation between the YAP/TAZ signals and levels of phosphorylated ERK (pERK). GSEA was performed with regard to high and low levels of pERK using the TCGA database. Barcode indicates gene positions and the y-axis indicates the extent of enrichment.

Supplementary Figure 3

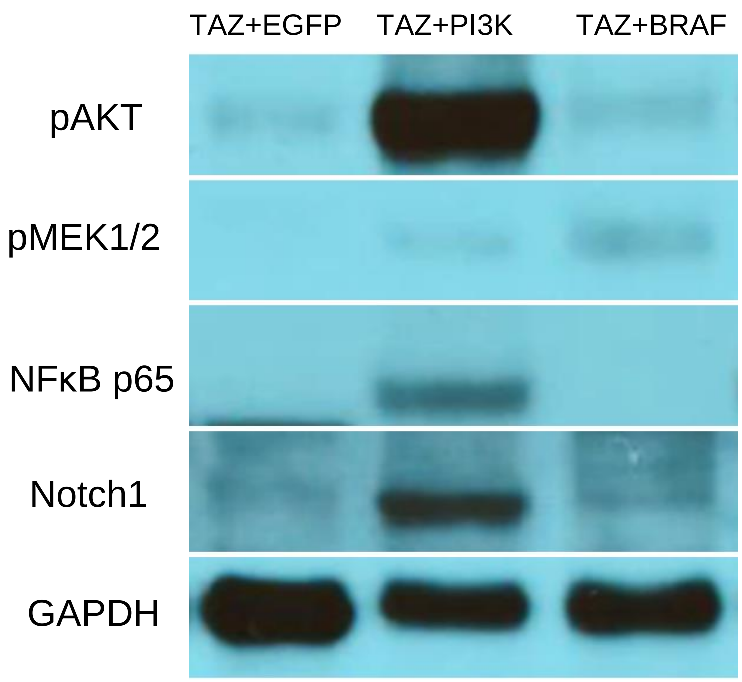

Supplementary Figure 3. Western blots showing expression levels of indicated genes in tumors from each group shown in Figure 5.
