# Supplementary material for: Activated TAZ induces liver cancer in collaboration with EGFR/HER2 signaling pathways
Source: BMC Cancer. 2022 Apr 19;22:423. doi: 10.1186/s12885-022-09516-1 (PMC9019950; doi:10.1186/s12885-022-09516-1)
Supplement: Supplementary file 2 — Additional file 2. [file 12885_2022_9516_MOESM2_ESM.docx]

**Supplementary Table 1. IHC positivity in human HCC and CC tissues**

| **IHC results** | **HCC** | **CC** |
| --- | --- | --- |
| **YAP/TAZ+** | 13/19 (68%) | 17/20 (85%) |
| pMEK1/2+ in YAP/TAZ+ | 11/13 (85%) | 12/17 (71%) |
| pAKT+ in YAP/TAZ+  pMEK1/2$-$ & pAKT$-$ in YAP/TAZ+  **YAP/TAZ**$\mathbf{-}$  pMEK1/2$-$ & pAKT$-$ in YAP/TAZ$-$ | 3/13 (23%)  2/13 (15%)  6/19 (32%)  6/6 (100%) | 15/17 (88%)  2/17 (12%)  3/20 (15%)  2/3 (67%) |
