# Supplementary material for: Activated TAZ induces liver cancer in collaboration with EGFR/HER2 signaling pathways
Source: BMC Cancer. 2022 Apr 19;22:423. doi: 10.1186/s12885-022-09516-1 (PMC9019950; doi:10.1186/s12885-022-09516-1)

The original western blot images for supplementary figure 3.

pAKT (cell signaling, #4060)
Phospho-MEK1/2 (Ser217/221) (cell signaling, #9154)
NFκB p65 (Santa Cruz, sc-372)
Activated Notch1 (Abcam, ab8925)
GAPDH (cell signaling, #2118)

1) EGFP
2) TAZ + EGFP
3) TAZ + PI3K
4) TAZ + Braf

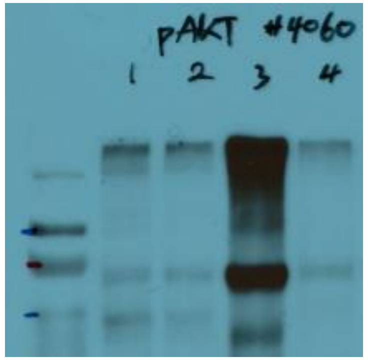

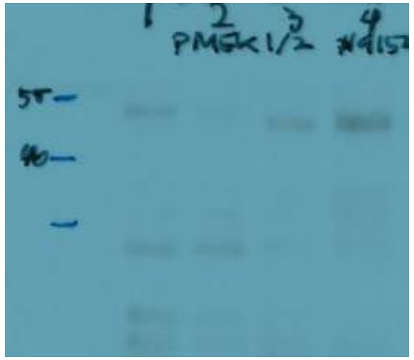

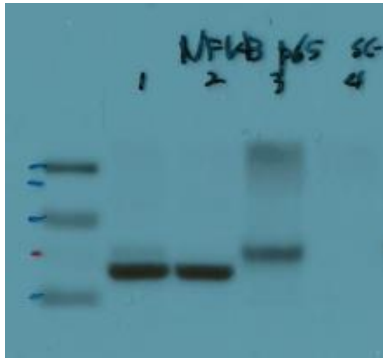

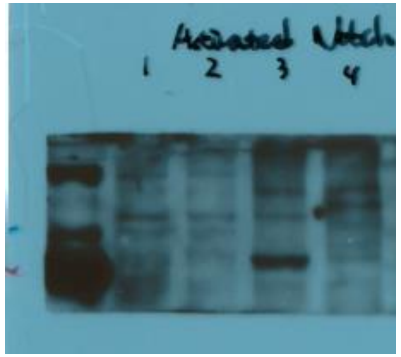

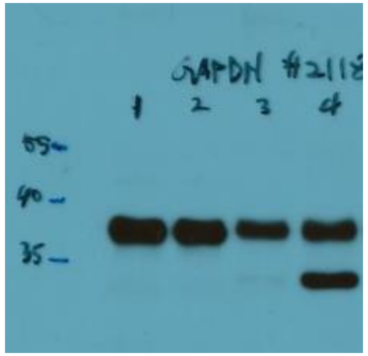

Supplement: Supplementary file 3 — Additional file 3. [file 12885_2022_9516_MOESM3_ESM.pdf]
